# Distinct Host and Bacterial Profiles in Murine Single Versus Co-Infection with Foal-Isolated *Streptococcus equi* and *Streptococcus dysgalactiae*

**DOI:** 10.3390/microorganisms14081674

**Published:** 2026-07-30

**Authors:** Zhen Wang, Zi-Xian Wang, Ge-Jin Lu, Li-Liang Xia, Guang-Hui Lu, Xue-Jun Guo, Wen-Can Hao, Yu-Jiao Zhang, Ru-Han Jiang, Shao-Jie Xu, Yan-Feng Li, Zi-Tong Shi, Zhong-Zheng Xu, Jun-Feng Qi, Zhi-Ying Shao, Jing Li, Ling-Wei Zhu, Lin Zheng

**Affiliations:** 1 School of Biological and Food Engineering, Huanghuai University, Zhumadian 463000, China; 2 Key Laboratory of Jilin Province for Zoonoses Prevention and Control, State Key Laboratory of Pathogen and Biosecurity, Academy of Military Medical Sciences, Changchun 130000, China; 3 Hebei Shuobo Animal Husbandry Service Co., Ltd., Tangshan 063500, China; vets2015@163.com; 4 College of Animal Sciences & Technology, Northwest A&F University, Yangling 712100, China; 5 Qinghai Center for Animal Disease Prevention and Control, Xining 810003, China; lijiingcom31960@163.com

**Keywords:** *Streptococcus equi*, *Streptococcus dysgalactiae*, co-infection, pan-genome, pulmonary microbiota, transcriptome

## Abstract

This study investigated the interactive pathogenic characteristics of co-infection with *Streptococcus equi* (*S. equi*) and *Streptococcus dysgalactiae* (*S. dysgalactiae*) isolated from diseased foals. Integrating genomic analysis, in vitro phenotypic assays, and a murine intranasal infection model, we comparatively analyzed the biological traits and pathogenic differences between single and dual infections. Comparative genomic analysis revealed divergent virulence profiles between the two isolates, and both species harbored open pan-genomes. In vitro, the biofilm biomass of strain F1 was higher than that of all other groups. No synergistic hemolytic activity was observed in co-cultures of the two isolates. In vivo infection results indicated that streptococcal infection significantly altered the structure and abundance of pulmonary microbiota in mice. Furthermore, single and dual infections displayed strain-specific and time-dependent microbial disparities at early and late infection stages, which were associated with differential enrichment of multiple immune- and metabolism-related Kyoto Encyclopedia of Genes and Genomes (KEGG) pathways, including the Tumor Necrosis Factor (TNF) and Mitogen-Activated Protein Kinase (MAPK) signaling pathways. Notably, co-infection triggered the most severe pulmonary microbiota dysbiosis and aberrant functional pathway regulation. This study preliminarily characterized the distinct phenotypic and pathological alterations induced by dual streptococcal infection, providing experimental evidence and novel insights for further exploring the interactive features of equine streptococcal mixed infections.

## 1. Introduction

*Streptococcus equi* (*S. equi*) is a Gram-positive, chain-forming facultative anaerobic coccus and the primary causative agent of equine strangles, one of the most prevalent and highly contagious infectious diseases affecting equine populations. As a typical hemolytic streptococcus, this pathogen exhibits stable β-hemolytic activity, robust epithelial adhesion capacity, and biofilm-forming potential, which facilitate its colonization and persistent survival in the equine respiratory tract. Clinically, equine strangles is characterized by suppurative lymphadenitis, high fever, and severe respiratory symptoms. Transmitted primarily through nasal secretions and contaminated environmental substrates, *S. equi* spreads rapidly among horse herds. Importantly, recovered horses frequently become asymptomatic long-term carriers, a unique epidemiological feature that markedly increases the risk of recurrent and persistent disease outbreaks [1]. As a subspecies of *Streptococcus equi subsp. zooepidemicus* can infect humans under specific conditions [2]. Numerous clinical cases have documented severe invasive infections in humans, including sepsis, meningitis, and endocarditis, associated with contact with infected horses or ingestion of unpasteurized, pathogen-contaminated dairy products [2,3,4].

*Streptococcus dysgalactiae* (*S. dysgalactiae*) is a multi-host pathogenic streptococcus with strong environmental adaptability and an extensive animal host spectrum. As a typical Gram-positive hemolytic *streptococcus*, it exhibits prominent hemolytic activity, potent biofilm-forming ability, and strong adhesion to host tissues. These intrinsic virulence traits are critical for its pathogenicity in animal hosts. This bacterium can trigger a wide spectrum of invasive disorders in susceptible animals, including skin and soft tissue infections, necrotizing fasciitis, toxic shock syndrome, and osteoarthritis [5]. To date, *S. dysgalactiae* isolates have been recovered from various livestock species, such as pigs, sheep, and cattle, reflecting its remarkable cross-host adaptability [6].

Although the pathogenic characteristics and health risks of single infections with *S. equi* and *S. dysgalactiae* in equine hosts have been well documented, shifts in bacterial phenotypic traits and host pathological responses induced by their co-infection remain poorly characterized. It remains unclear whether co-infection reshapes hemolytic activity and biofilm formation of the two strains, as well as how their interaction modulates pulmonary pathogenicity and disrupts host immune homeostasis. Systematic profiling of phenotypic and transcriptomic alterations driven by dual infection is currently lacking. In this study, *S. equi* and *S. dysgalactiae* were co-isolated from a single diseased foal in 2016. To fill this research gap, we integrated genomic analysis, in vitro phenotypic assays, and an in vivo murine intranasal infection model to systematically characterize the bacterial pathogenic and host pathological differences between single and dual streptococcal infections.

## 2. Materials and Methods

### 2.1. Sampling and Bacterial Identification

In 2016, eleven 6–7-month-old newly imported foals at a horse farm in China developed typical respiratory clinical signs including inappetence and nasal discharge, within two weeks after arrival. One foal displayed obvious submandibular lymphadenectasis. Most foals achieved partial clinical relief after three days of intravenous antimicrobial treatment without full recovery. By contrast, the foal with submandibular swelling exhibited progressive deterioration, accompanied by mandibular ulceration and recumbency. To isolate and identify the causative pathogens, purulent exudate samples were collected from the ulcerative lesions of this severely diseased foal using sterile cotton swabs. Samples were transferred into sterile containers filled with normal saline, transported to the laboratory under refrigeration immediately, and subjected to bacterial isolation and identification.

Upon arrival at the laboratory, samples were streaked onto 5% sheep blood Todd-Hewitt agar (THA) plates and incubated aerobically at 37 °C overnight. Isolates were initially distinguished by colonial morphology, and species-level identification was further validated via 16S rRNA gene sequencing. The universal bacterial primer pair 12F (AGAGTTTGATCMTGGCTCAG) and 1492R (TACGGYTACCTTGTTACGACTT) was used for 16S rRNA gene amplification. The PCR cycling conditions were as follows: initial denaturation at 94 °C for 5 min, followed by 30 cycles of denaturation at 94 °C for 45 s; annealing at 55 °C for 45 s; and extension at 72 °C for 90 s; followed by a final extension step at 72 °C for 10 min. Confirmed *Streptococcus* isolates were suspended in brain heart infusion (BHI) medium supplemented with 50% glycerol and stored at −80 °C for follow-up experiments.

### 2.2. Antimicrobial Susceptibility Testing

The Kirby–Bauer (K-B) disk diffusion method were performed to determine the antimicrobial susceptibility profiles of the five isolates against 11 antimicrobial agents: tetracycline (TE: 30 μg), gentamicin (CN: 10 μg), chloramphenicol (C: 30 μg), meropenem (MEM: 10 μg), ampicillin (AMP: 10 μg), tigecycline (TGC: 15 μg), sulfisoxazole (SXT: 25 μg), ciprofloxacin (CIP: 5 μg), amikacin (AK: 30 μg), cefotaxime (CTX: 30 μg), and fosfomycin (FOT: 200 μg). All K-B susceptibility disks were purchased from OXOID (Basingstoke, UK), and these antimicrobials represent the primary agents routinely used for clinical therapy at the target horse farm:

Single colonies of each test strain were picked from 5% sheep blood THA, inoculated into Mueller-Hinton (MH) broth, and incubated at 37 °C for 16 h. The resultant bacterial suspension was diluted with sterile MH broth and adjusted to a 0.5 McFarland turbidity standard. A sterile cotton swab was dipped into the standardized suspension and used to uniformly inoculate the surface of MH agar plates supplemented with 5% sheep blood. The 11 antimicrobial disks were gently placed onto the dried agar surface using sterile forceps, with a maximum of five disks evenly distributed on each plate. Plates were inverted and incubated at 37 °C for 16 h. Following incubation, the diameter of each zone of inhibition was measured using a vernier caliper. Zone diameters were interpreted as susceptible, intermediate, or resistant in accordance with the Clinical and Laboratory Standards Institute (CLSI) M100-2024 [7] breakpoints for β-hemolytic *streptococci*. *Streptococcus pneumoniae* (*S. pneumoniae*) ATCC 49619 served as the quality control strain for each batch of tests to ensure data reliability. All experiments were performed in three independent biological replicates.

### 2.3. Bacterial Genome Analysis

#### 2.3.1. Genome DNA Extraction and Whole-Genome Sequencing

Genomic DNA was extracted using a bacterial genomic DNA extraction kit (Omega: Norcross, GA, USA). DNA concentration and purity were quantified with a Qubit 2.0 fluorometer (Thermo Scientific: Waltham, MA, USA). Sequencing libraries were constructed with the NEBNext Ultra DNA Library Prep Kit for Illumina (NEB: Ipswich, MA, USA) according to the manufacturer’s instructions, and unique index barcodes were added to distinguish different samples. Briefly, genomic DNA was fragmented by sonication to an average insert size of 350 bp. DNA fragments were subjected to end repair, A-tailing, and adapter ligation with full-length Illumina adapters, followed by PCR enrichment. PCR products were purified with the AMPure XP system. Library fragment size distribution was assessed on an Agilent 2100 Bioanalyzer, and library quantification was conducted via quantitative real-time PCR. Whole-genome paired-end sequencing (PE150) was performed on the Illumina NovaSeq platform at Beijing Novogene Bioinformatics Technology Co., Ltd.: Beijing, China.

#### 2.3.2. Genome Assembly and Comparative Genomic Analyses

Pair-wise average nucleotide identity (ANI) analysis was conducted using the EzBioCloud OrthoANI tool (http://www.ezbiocloud.net/tools/ani (accessed on 8 January 2021)) to compare the two *de novo* assembled genomes against two publicly available reference genomes: *S. dysgalactiae* 159 (NZ_AP023394.1) and *S. equi* SEZ33 (NZ_CP065061.1) [8]. Functional annotation against the NCBI non-redundant (NR) protein database was further performed to support species identification. ANI values ≥ 95% were considered the threshold for species demarcation, following commonly used criteria for prokaryotic species boundaries. Open reading frames (ORFs) were predicted and annotated using the RASTtk pipeline (v2.0) [9,10]. Additional functional annotation was performed by BLASTP and BLASTN searches against the NCBI NR database. To identify virulence factors and acquired antimicrobial resistance genes, sequence comparisons were conducted against the Virulence Factor Database (VFDB) (https://www.mgc.ac.cn/VFs/ (accessed on 8 January 2021)) databases and the Comprehensive Antibiotic Resistance Database (CARD) (https://card.mcmaster.ca (accessed on 8 January 2021)) using BLAST with an identity threshold of ≥40% and coverage ≥ 40% [11,12]. The allelic profiles of seven housekeeping genes and STs (sequence types) of the strains F1–F5 were retrieved by submitting their genome assemblies to MLSTFinder (https://genepi.food.dtu.dk/mlstfinder (accessed on 8 January 2021)).

Comparative genomic analyses included genomic synteny analysis, classification of core and strain-specific genes, and phylogenetic reconstruction based on gene families. Whole-genome alignment between the test isolates and reference strains was performed using MUMmer and LASTZ [13]; an additional dataset comprising 19 *S. dysgalactiae* and 18 *S. equi* genomes was included for expanded comparison (information on reference strains is shown in Supplemental Table S1). Genomic synteny patterns were visualized based on the alignment outputs. Core and strain-specific genes were classified via homologous protein clustering using CD-HIT v4.6.1 with a cutoff of 70% amino acid identity and a minimum sequence length of 110 bp. A heatmap was generated to visualize accessory genome profiles. Single-copy core proteins identified from the core/pan-genome analysis were multiple-aligned using MUSCLE. The resulting alignments were used to construct a neighbor-joining (NJ) phylogenetic tree via MEGA.

### 2.4. Biofilm Formation and Hemolytic Characteristics of S. dysgalactiae and S. equi

Overnight cultures of *S. dysgalactiae* and *S. equi* were separately sub-cultured in MH broth. The turbidity of each bacterial suspension was adjusted to 0.5–0.6 McFarland using a densitometer, followed by a 100-fold dilution with fresh MH broth. A 200 μL aliquot of the diluted suspension was transferred into each well of a 96-well plate, and the plates were incubated at 37 °C for 20 h. After incubation, the culture supernatants were aspirated, and each well was washed three times with 300 μL of sterile PBS (phosphate-buffered saline). Methanol was added to fix biofilm biomass for 15 min; after methanol was discarded, plates were air-dried at room temperature. Fixed biofilms were stained with 200 μL of 0.1% (*w*/*v*) crystal violet solution for 15 min. Excess strain was removed by rinsing with distilled water, and plates were rinsing with distilled water, and plates were air-dried again. After two additional PBS washes (300 μL per well), 200 μL of 75% ethanol was added to elute the bound crystal violet, and the plates were incubated for 15 min. Wells containing only PBS without bacteria were set as negative controls. All assays were conducted in three independent biological replicates, and absorbance at 600 nm (OD_600_) was measured using a microplate reader. Biofilm production capacity was classified according to the threshold criteria described previously [14]. The cutoff OD value (ODc) was calculated as the mean OD_600_ of negative controls plus three standard deviations, and biofilm-forming capacity was categorized as follows:

No biofilm-forming ability: OD ≤ ODc;

Weak biofilm-forming ability: ODc < OD ≤ 2ODc;

Moderate biofilm-forming ability: 2ODc < OD ≤ 4ODc;

Strong biofilm-forming ability: OD > 4ODc.

To characterize hemolytic phenotypes and interbacterial interactions between the two strains, an adjacent parallel streaking assay was carried out. Briefly, *S. dysgalactiae* and *S. equi* were streaked in parallel on 5% sheep blood THA plates with a 1 mm spacing between the two streaks. Plates were incubated at 37 °C for 24 h, after which hemolytic zone morphology at the streak junction and phenotype interactions within the contact area were visually observed.

### 2.5. Virulence Assay

#### 2.5.1. Bacterial Preparation and Quantification

Mid-logarithmic-phase cultures of *S. dysgalactiae* and *S. equi* were serially diluted 10-fold in PBS from 10^−1^ to 10^−9^. Aliquots of each dilution were plated onto 5% sheep blood THA plates for colony-forming units (CFU) enumeration to determine inoculum concentrations for subsequent experiments. Plates were incubated at 37 °C for 12–16 h, and colonies were counted on plates containing 30–300 colonies. Bacterial concentrations were calculated using Formula (1):

(1) CFU/mL = (Average colony count × Dilution factor)/plated Volume

#### 2.5.2. LD_50_ Determination

Considering the prohibitive cost, strict ethical supervision and operational limitations of equine infection experiments, 4-week-old female BALB/c mice were selected as a recognized surrogate animal model for equine streptococcal respiratory infection. Mice were purchased from Baijing Gudu Jintai Biotechnology Co., Ltd.: Beijing, China. and housed in a SPF (specific pathogen-free) facility under standardized conditions: a 12 h light/dark cycle, temperature of 22–25 °C, and relative humidity of 50–60%. Five mice were housed per cage with free access to sterilized feed and water. All animal procedures were approved by the Laboratory Animal Welfare and Ethics Committee of the Institute of Military Veterinary Science, Academy of Military Medical Sciences (IACUC of AMMS-11-2025-043) and were conducted in accordance with the ARRIVE guidelines.

To determine the LD_50_, mice were randomly assigned to ten groups (*n* = 10 per group) using a random number table, including nine bacterial challenge groups and one PBS negative control group. Mice received intraperitoneal injections of serially diluted suspensions of *S. dysgalactiae* (1 × 10^5^–1 × 10^8^ CFU per mouse) or *S. equi* (1 × 10^6^–1 × 10^9^ CFU per mouse), with an infection volume of 0.1 mL per mouse.

#### 2.5.3. Intranasal Infection Model

Intraperitoneal LD_50_ assays were first conducted to preliminarily compare strain virulence; referring to these virulence data, gradient-dose intranasal infection pretests were carried out, and a standardized challenge dose was finally determined to establish a mouse intranasal model featuring severe acute pulmonary injury. The study aimed to induce robust and observable lung pathology within the observation window; therefore, relatively high bacterial loads were selected for intranasal instillation rather than sublethal doses used for mild infection models.

Mice were randomly divided into nine treatment groups (*n* = 5 per group): (1) TG1: Day 1 single *S. dysgalactiae* infection (1 × 10^9^ CFU/mL); (2) TG3: Day 3 single *S. dysgalactiae* infection (1 × 10^9^ CFU/mL); (3) TG7: Day 7 single *S. dysgalactiae* infection (1 × 10^9^ CFU/mL); (4) MG1: Day 1 single *S. equi* infection (1 × 10^8^ CFU/mL); (5) MG3: Day 3 single *S. equi* infection (1 × 10^8^ CFU/mL); (6) MG7: Day 7 single *S. equi* infection (1 × 10^8^ CFU/mL); (7) HG1: Day 1 dual-strain co-infection (10:1 CFU ratio of *S. dysgalactiae* to *S. equi*); (8) HG3: Day 3 dual-strain co-infection (10:1 CFU ratio of *S. dysgalactiae* to *S. equi*); (9) HG7: Day 7 dual-strain co-infection (10:1 CFU ratio of *S. dysgalactiae* to *S. equi*); and sterile PBS control. Each mouse received a total volume of 50 μL of bacterial suspension via intranasal instillation under light isoflurane anesthesia. Lung tissue samples were harvested at 1, 3, and 7 days post-infection. Due to the nature of the infection procedure, blinding of the investigators during inoculation was not feasible, but outcome assessments (e.g., cytokine measurement) were performed by personnel blinded to group assignment where possible.

#### 2.5.4. Sample Collection and Cytokine Measurement

Mice were anesthetized with 4–5% isoflurane for 3–5 min until deeply anesthetized. Blood was collected from the retro-orbital venous plexus, after which all animals were humanely euthanized by cervical dislocation. Whole blood was allowed to clot at room temperature for at least 1 h and then centrifuged sequentially at 4000 rpm (4 °C, 20 min) and 8000 rpm (4 °C, 5 min) to obtain serum. Serum levels of TNF-α, MCP-1, IFN-γ, IL-6, and IL-1β were measured using commercial enzyme-linked immunosorbent assay (ELISA) kits (Colorful Gene Biological Technology: Wuhan, China). Absorbance at 450 nm was measured within 15 min after addition of stop solution, and cytokine concentrations were calculated by four-parameter logistic curve fitting using ELISA Calc software (v 0.2).

#### 2.5.5. Tissue Harvesting and Bacterial Load Quantification

After euthanasia, mice were surface-disinfected with 75% ethanol, and the thoracic cavities were opened via a midline incision to isolate intact lung tissues. Gross pathological lesions were photographed and recorded. From each individual lung, three tissue fragments (approximately 3 mg each) were collected: two fragments were snap-frozen in liquid nitrogen and stored independently for subsequent transcriptomic and pulmonary microbiota sequencing. Multi-omics analysis was performed using these individual tissue samples collected at Days 1 and 7 post-infection, thereby providing biological replicates for omics profiling. The third tissue fragment from each mouse was used for pulmonary bacterial load quantification.

For bacterial load quantification, lung tissues from five mice within the same group were pooled and homogenized as a single composite sample to ensure sufficient homogenate volume for reliable colony counting. This pooling strategy precluded the use of individual biological replicates for CFU analysis; therefore, standard deviation calculation and intergroup statistical comparisons were not performed for pulmonary bacterial load data.

Pooled lung homogenates were prepared in 2 mL of sterile 0.9% saline, serially diluted, and plated onto 5% sheep blood THA plates. After overnight incubation at 37 °C, colonies were counted, and pulmonary bacterial loads were calculated and expressed as log_10_-transformed CFU per gram of lung tissue. Data visualization was performed using R software (v.4.2.2) with the ggplot2 package (v.3.4.2).

### 2.6. Transcriptome Sequencing of Murine Lung Tissues

In this study, lung tissue samples collected at Day 1 post-infection (early infection stage) and Day 7 post-infection (mid-to-late infection stage) were subjected to transcriptome sequencing and comparative analysis. Samples harvested at Day 3 post-infection, which represented a shorter time interval between the two selected time points, were excluded from sequencing. Total RNA was extracted from murine lung tissues using the RNAprep Pure Cell/Bacteria Kit (TIANGEN: Beijing, China) according to the manufacturer’s instructions. Each experimental group included five infected mice, among which three individuals with consistent pulmonary pathological phenotypes were selected as independent biological replicates for transcriptome sequencing. Poly(A) mRNA was enriched via oligo(dT) selection, and sequencing libraries were constructed and subjected to paired-end sequencing on the Illumina NovaSeq platform.

Raw sequencing reads were quality-filtered to generate clean reads, which were aligned to the *Mus musculus* GRCm39 reference genome (GenBank assembly accession: GCF_000001635.27) using HISAT2. Reads with mapping quality < 10, unpaired reads, and multi-mapped reads were discarded prior to gene quantification. Gene-level read counts were calculated using featureCounts from the Subread package based on the corresponding gene annotation file. differentially expressed genes (DEGs) were identified using the DESeq2 R package (v1.38.3), with thresholds set as |log2(FoldChange)| ≥ 1 and padj ≤ 0.05.

Transcriptional patterns of core immune-related genes were visualized using R (v.4.2.2) and the ggplot2 package (v3.4.2). The analyzed gene set included pro-inflammatory mediators (IL-6, IFN-γ, TNF-α, CCL2/MCP-1, CCL12, IL-1α, IL-1β), corresponding receptors (IL-6R, IL-6ST, IFNGR1, IFNGR2, TNFR1, TNFR2, CCR2, IL-1R1, IL-1RAP, IL-1R2), the IL-1 receptor antagonist IL-1RN, and immunosuppressive markers (ARG1, CTLA-4, HAVCR2, PD-1, S100A9, CD163, FOXP3, IL-10, S100A8, TGF-β1).

### 2.7. Metagenomic Sequencing of Murine Lung Tissue

#### 2.7.1. Total DNA Extraction and Metagenomic Sequencing

In this study, lung tissue samples collected at Day 1 post-infection (early infection stage) and Day 7 post-infection (mid-to-late infection stage) were subjected to transcriptome sequencing and comparative analysis. Samples harvested at Day 3 post-infection, which represented a shorter time interval between the two selected time points, were excluded from sequencing. Total DNA was extracted from murine lung tissues using the TIANGEN Blood/Cell/Tissue Genomic DNA Extraction Kit according to the manufacturer’s protocols. Each experimental group contained five infected mice. Among them, three individuals with consistent pulmonary pathological lesions were selected as three independent biological replicates for metagenomic sequencing. Purified genomic DNA was sheared to an average insert size of 350 bp using a Covaris ultrasonicator for Illumina PE150 library construction. All libraries were quantified by qPCR, and only libraries with a concentration above 3 nM were used for high-throughput sequencing.

Raw reads were quality-trimmed and filtered using fastp (v0.23.1). Host-derived mouse sequences were removed by aligning clean reads against the *Mus musculus* reference genome (GRCm39) using Bowtie2 (v2.4.5). The remaining high-quality reads were de novo assembled using MEGAHIT (v1.2.9), and open reading frames (ORFs) were predicted using MetaGeneMark (v2.1). Redundant gene sequences were clustered and removed with CD-HIT (v4.5.8) to construct an NR gene catalog. Gene abundance was quantified by mapping clean reads to the NR gene catalog via Bowtie2. Genes with low abundance (≤2 mapped reads per sample) were excluded for subsequent analyses to minimize technical noise.

#### 2.7.2. Microbial Taxonomic Composition and Functional Annotation

For taxonomic profiling of microbial communities, unigenes were aligned against the NCBI NR protein database using DIAMOND (v2.1.9) [15]. Taxonomic assignments were generated via the Lowest Common Ancestor (LCA) algorithm implemented in MEGAN 6 (v6.21.6) [16]. Genus-level microbial composition and relative abundance were calculated, and community distribution patterns were visualized using heatmaps generated in R (v.4.2.2).

For functional analysis, unigenes were annotated against the Kyoto Encyclopedia of Genes and Genomes (KEGG) database using DIAMOND (v2.1.9) to obtain KEGG Orthology (KO) information. The relative abundance of KO entries was quantified and visualized as heatmaps using R (v.4.2.2) [17]. For differential abundance analysis of microbial taxa and functional pathways, the Benjamini–Hochberg false discovery rate (BH-FDR) correction was applied to correct all raw *p*-values. Only taxa and pathways with FDR-adjusted *p* < 0.05 were considered statistically significant.

### 2.8. Statistical Analysis

Normality and homogeneity of variance tests were carried out on all quantitative data, including biofilm OD values and serum cytokine concentrations. An unpaired t-test was adopted for comparisons between two groups, while one-way ANOVA combined with Tukey’s multiple comparison test was used for multi-group comparisons. The threshold for statistical significance was set at *p* < 0.05. All graphs, heatmaps and phylogenetic trees were visualized by R software (v4.2.2) based on ggplot2 and related bioinformatics tools. All in vitro and in vivo phenotypic experiments were set up with at least three independent biological replicates.

### 2.9. Nucleotide Sequence Accession Numbers

The complete sequence of *S. dysgalactiae* isolates (F1, F2, F3) and *S. equi* (F4 and F5) has been submitted to GenBank under the accession numbers JBWKAG000000000, JBWKAI000000000, JBWKAH000000000, JBWKAE000000000, and JBWKAF000000000. Transcriptome and metagenome data derived from the lungs of mice in different infection groups at distinct post-infection stages have been uploaded to the CNCB (China National Center for Bioinformatics) (https://ngdc.cncb.ac.cn/gsub accessed on 20 January 2026) under accession numbers CRA037801 and CRA038013.

## 3. Results

### 3.1. Isolation and Identification of S. dysgalactiae and S. equi

Samples were streaked onto 5% sheep blood THA plates for isolation, and two types of colonies with markedly distinct morphological characteristics were recovered. The first colony type was 1 mm in diameter, white and circular with smooth intact margins, and exhibited α-hemolysis (incomplete hemolysis); three isolates designated F1, F2, and F3 belonged to this type. The second colony type shared similar basic morphological features but reached 2 mm in diameter and displayed β-hemolysis (complete hemolysis), and comprised two isolates designated F4 and F5. Based on 16S rRNA gene sequencing (sequences provided in Supplementary Table S2), isolates F1, F2, and F3 were preliminarily identified as *S. dysgalactiae*, whereas F4 and F5 were identified as *S. equi*. Antimicrobial susceptibility testing was performed via the K-B (Kirby–Bauer) disk diffusion method. The results revealed that all isolates were susceptible to all tested antimicrobial agents, with no resistance phenotypes detected (Table 1).

### 3.2. Genomic Characterization of S. dysgalactiae and S. equi

To achieve precise species delineation, genomic DNA was extracted from the isolates, and their draft genome sequences were subsequently determined. Genome assembly statistics of five *Streptococcus* strains are shown in Supplementary Table S3. Species identification was verified via ANI analysis. ANI values were calculated against the reference strain *S. dysgalactiae* 159 (NZ_AP023394.1), yielding values of 97.27%, 97.35%, and 97.32% for F1, F2, and F3, respectively. Against the reference genome of *S. equi* SEZ233 (NZ_CP065061.1), the ANI values for F4 and F5 were 96.94% and 96.93%, respectively. Multilocus sequence typing (MLST) analysis identified seven housekeeping gene loci (*atoB, gki, gtr, murI, mutS, recP, xpt*) from the genomes of F1, F2, and F3 (detailed MLST data in Supplementary Table S4). Isolate F1 belonged to STs 610, while F2 and F3 were assigned to ST611. Seven housekeeping loci (*arcC, nrdE, proS, spi, tdk, tpi, yqiL*) were identified from the genomes of F4 and F5 (Supplementary Table S4), and both isolates were assigned to ST24.

Antibiotic resistance gene screening was performed using the CARD. The results revealed that *S. dysgalactiae* strains F1, F2, and F3 only harbored the efflux pump gene *lmrP*, while no putative antibiotic resistance genes were detected in *S. equi* strains F4 and F5. Virulence factor prediction was conducted using the VFDB. The two *S. equi* isolates F4 and F5 exhibited identical virulence factor profiles. In contrast, obvious differences were observed in the virulence profiles of the three *S. dysgalactiae* isolates; F2 and F3 shared identical profiles, whereas F1 displayed a distinct pattern. Specifically, strain F1 uniquely harbored putative dextran-binding protein, 2-methylisocitrate lyase, exotoxin G variant 8, and endoglycosidase, which were absent in F2 and F3. Conversely, several putative virulence factors detected in F2 and F3 were not identified in F1, including putative mitogenic factor MF4, MRP protein, hyaluronoglucosaminidase HylP, δ-1-pyrroline-5-carboxylate synthase (δ-glutamate-1-semialdehyde 2,1-aminomutase), porphobilinogen synthase, and porphobilinogen deaminase carried by strains F2 and F3, which were not detected in strain F1 (Supplementary Table S5).

### 3.3. Core/Pan-Genome Analysis of S. dysgalactiae and S. equi

**Figure 1:**
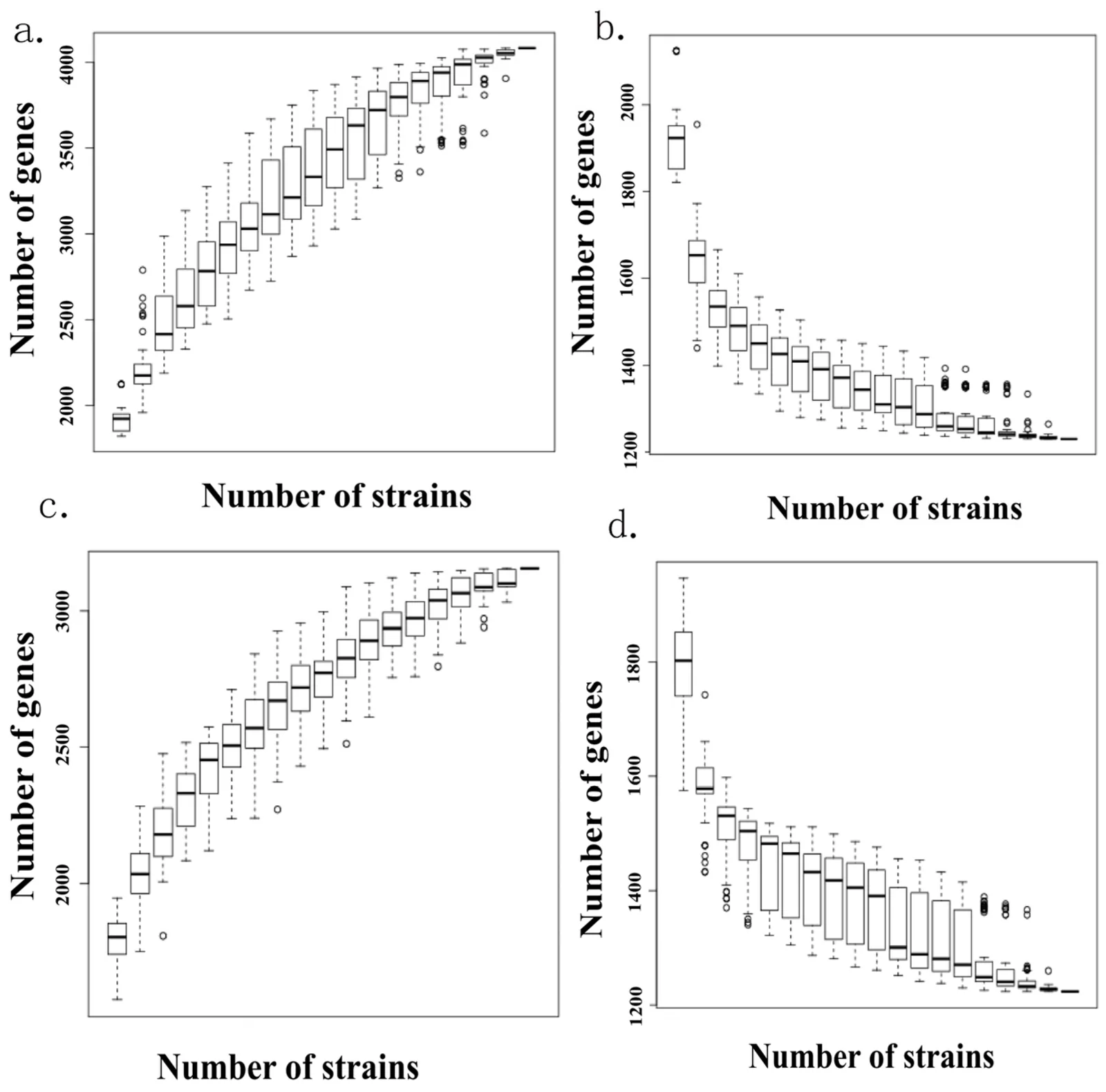
Core/pan-genome diversity curves: (**a**) the pan dilution curve *S. dysgalactiae*; (**b**) the core dilution curve *S. dysgalactiae*; (**c**) the pan dilution curve *S. equi*; (**d**) the core dilution curve *S. equi*.

To elucidate the genomic characteristics of *S. dysgalactiae* and *S. equi*, comparative genomic analyses were performed based on the five isolates obtained in this study (F1–F3 for *S. dysgalactiae* and F4–F5 for *S. equi*) and publicly available reference genomes. Pan-genome analysis indicated that both species harbored typical open pan-genomes, in which the core genome size gradually plateaued, whereas the pan-genome size continuously increased with the addition of new genomic datasets (Figure 1a,b). In total, the *S. dysgalactiae* pan-genome contained 4082 genes (1230 core and 2852 dispensable genes), while the *S. equi* pan-genome consisted of 3156 genes (1224 core and 1932 dispensable genes) (Figure 1c,d).

**Figure 2:**
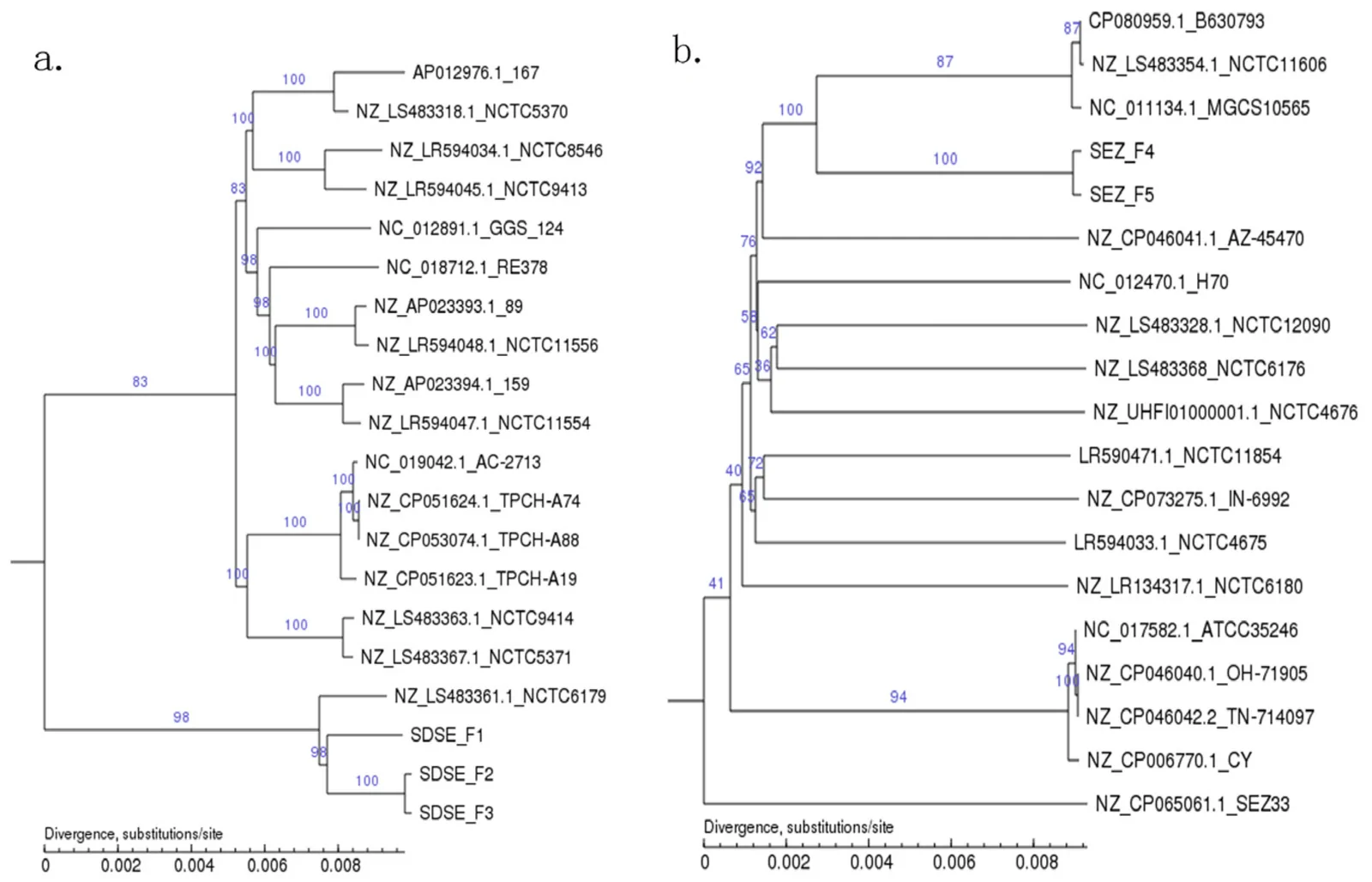
Core/pan-genome phylogenetic trees. (**a**) The core/pan-genome phylogenetic analysis of *S. dysgalactiae*. (**b**) The core/pan-genome phylogenetic analysis of *S. equi*.

**Figure 3:**
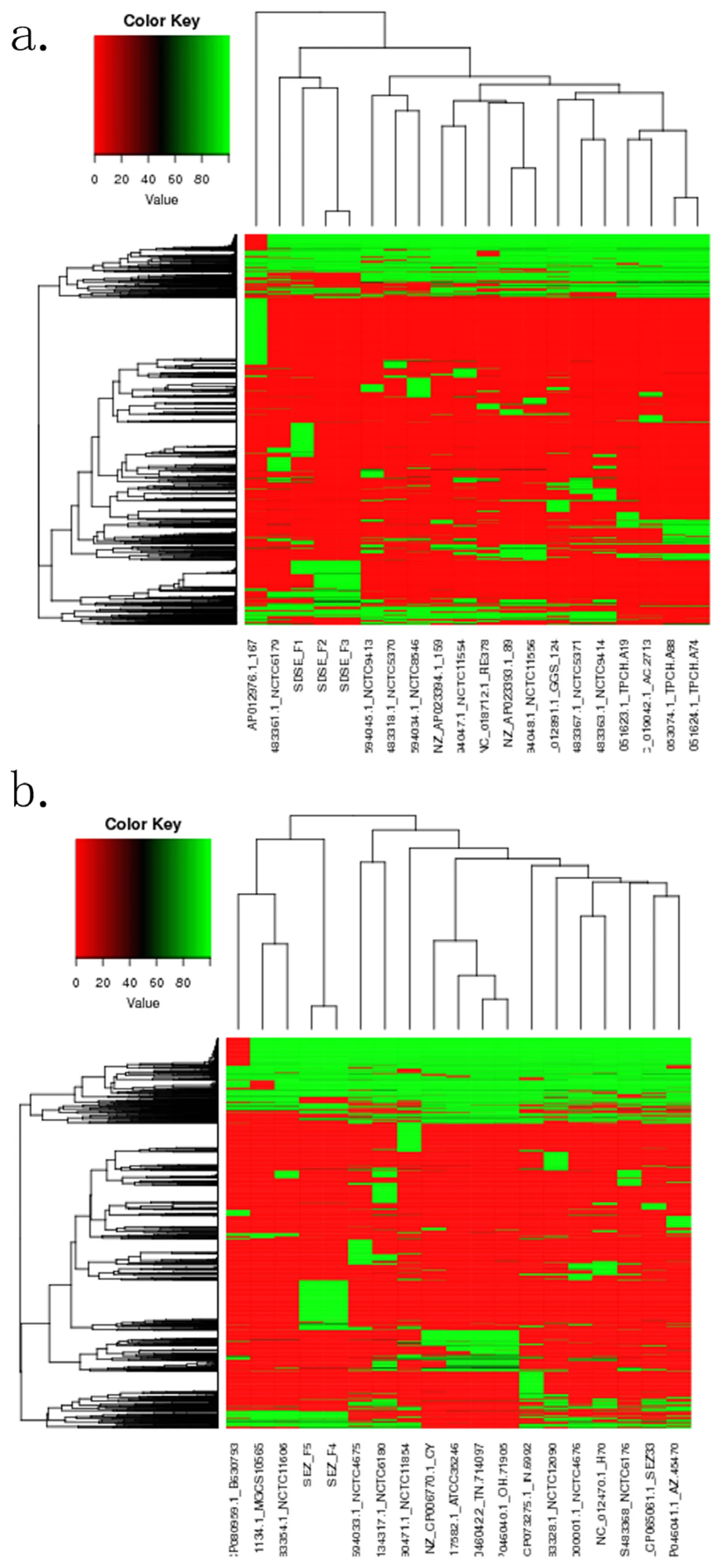
Dispensable heatmaps. (**a**) The dispensable heatmap of *S. dysgalactiae*. (**b**) The dispensable heatmap of *S. equi*.

A phylogenetic tree constructed based on core-genome alignment demonstrated that the three *S. dysgalactiae* isolates (F1, F2, and F3) clustered closely with the reference strain NCTC6179 (NZ_LS48336.1) (Figure 2a). Within this clade, strain F2 and F3 formed a subcluster distinct from F1. Consistent with this phylogenetic relationship, F2 and F3 exhibited highly similar dispensable gene profiles, which were remarkably different from those of F1 (Figure 2b). For *S. equi*, isolates F4 and F5 shared nearly identical dispensable gene patterns, although F4 harbored four strain-specific genes, compared to only two in F5 (Figure 3a,b).

### 3.4. Biofilm Formation and Hemolytic Activity of S. dysgalactiae and S. equi

To explore the synergistic or antagonistic pathogenic effects between *S. dysgalactiae* and *S. equi* during co-infection, representative strains were selected for phenotypic and functional verification. All five isolates (F1–F3 of *S. dysgalactiae* and F4–F5 of *S. equi*) were recovered from the same lesion of a single diseased foal. Phylogenetic and pan-genome analyses revealed limited intraspecies genetic variation among the three *S. dysgalactiae* isolates (F1, F2, and F3) and between the two *S. equi* isolates (F4 and F5), with only minor genomic polymorphisms and a small number of strain-specific genes identified. Accordingly, *S. dysgalactiae* F1 and *S. equi* F4 were selected as representative strains for subsequent phenotypic assays and co-infection experiments, while F2, F3, and F5 were retained solely for comparative genomic analysis to avoid redundant phenotypic validation.

The biofilm-forming capacities of monocultured and co-cultured strains were evaluated by measuring the optical density at 600 nm (OD). The normalized biofilm formation values were as follows: monocultured *S. dysgalactiae* F1 (0.32 ± 0.06), monocultured *S. equi* F4 (0.187 ± 0.04), co-cultured groups with F1:F4 ratios of 10:1 (0.164 ± 0.02) and 1:10 (0.149 ± 0.03), and ODc was 0.06. Except for *S. dysgalactiae* F1, which exhibited strong biofilm-forming capacity (OD > 4ODc), all other culture groups displayed moderate biofilm-forming ability (ODc < OD < 2ODc). Hemolytic activity assays showed that monocultured *S. dysgalactiae* F1 exhibited α-hemolysis, while *S. equi* F4 displayed typical β-hemolysis. After co-cultivation of the two strains at the above ratios, no changes in hemolytic phenotypes were observed, indicating that interspecific co-culture did not alter the hemolytic characteristics of either strain.

### 3.5. Mouse LD_50_ Result

Intraperitoneal injection of 0.1 mL of serially diluted *S. dysgalactiae* F1 suspensions induced dose-dependent mortality. At 10^9^ CFU per mouse, mortality reached 90% by Day 1 and 100% by Day 2. At 10^8^ CFU per mouse, mortality was 90% on Day 3 and 100% on Day 4. In contrast, 10^7^ and 10^6^ CFU per mouse showed mild lethality, with mortality rates of 10% on Day 3 and 20% on Day 4; no additional deaths occurred after Day 4.

Intraperitoneal challenge with serially diluted *S. equi* F4 yielded distinct lethal kinetics. The 10^8^ CFU per mouse achieved 100% mortality as early as Day 1. For the 10^7^ CFU per mouse, mortality climbed to 90% on Day 2 and reached 100% on Day 3. Animals receiving 10^6^ CFU per mouse suspensions showed progressive mortality: 10% on Day 3, 20% on Day 4, and 30% on Day 5; no additional deaths were recorded thereafter. The 10^5^ CFU per mouse recorded 10% mortality on Day 3 and 20% on Day 4, with no additional deaths observed for the remainder of the observation window.

### 3.6. Mouse Lung Infection Assay Results

**Figure 4:**
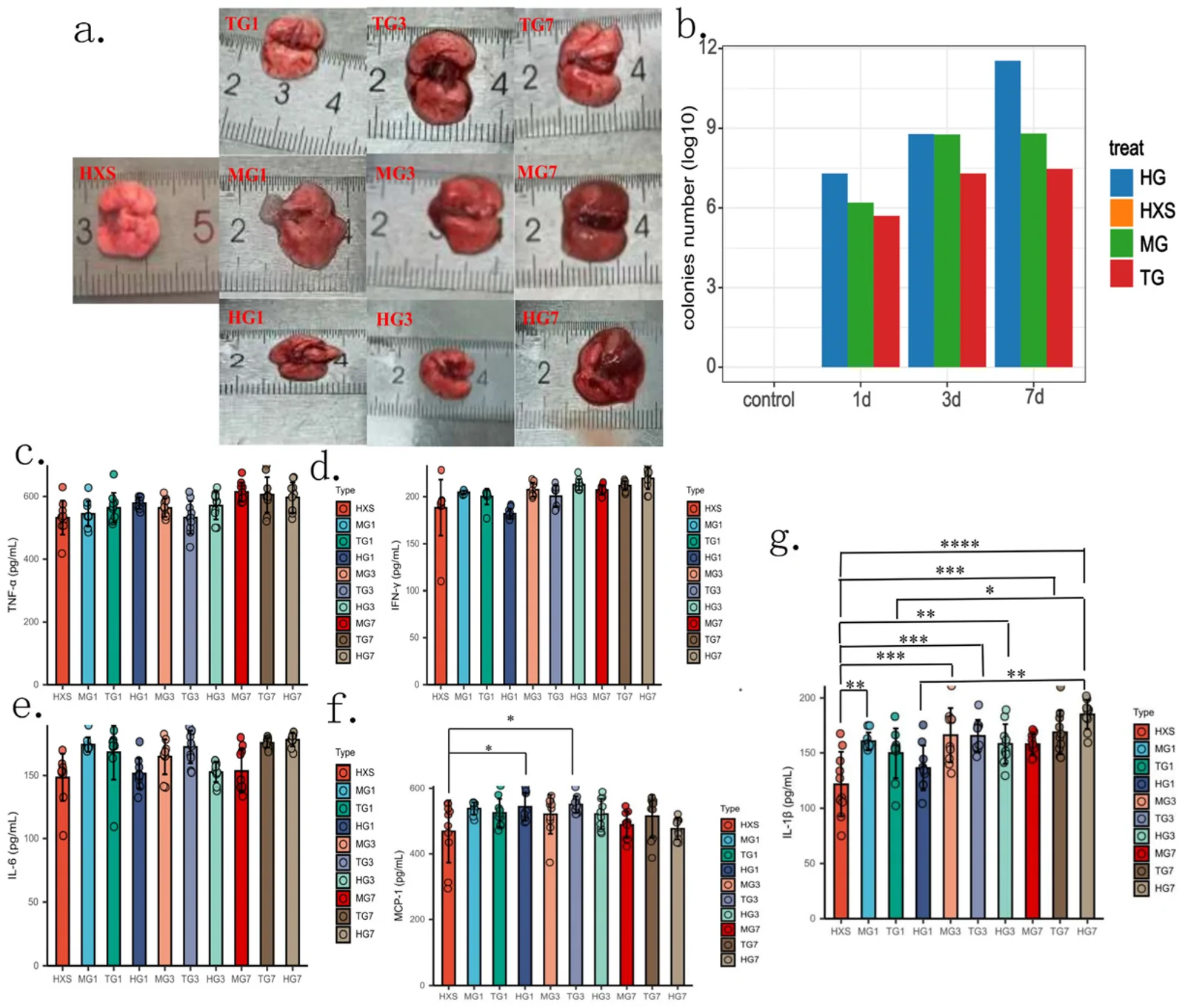
The pathological manifestations (**a**), colony counts (**b**), and immune factor levels (**c**–**g**) in mice infected with *S. dysgalactiae*, *S. equi* and their co-infection. HXS represents the lung tissues from uninfected healthy mice. TG1, TG3, and TG7 indicate lung tissues from mice singly infected with *S. dysgalactiae* at 1, 3, and 7 days post-infection, respectively. MG1, MG3, and MG7 correspond to mice with monoinfection of *S. equi* at 1, 3, and 7 days post-infection. HG1, HG3, and HG7 refer to mice co-infected with *S. dysgalactiae* and *S. equi* at 1, 3, and 7 days post-infection. For pathological observation and cytokine detection, tissues from five individual mice per group were analyzed independently (*n* = 5). For bacterial load quantification (**b**), lung tissues from five mice within each group were pooled prior to CFU enumeration; therefore, these CFU data were only interpreted descriptively without formal statistical comparison. For immune factor levels (**c**–**g**): an unpaired t-test was used for two-group comparisons, and one-way ANOVA followed by Tukey’s multiple comparison test was applied for multi-group comparisons of pathological and cytokine data. Statistical significance was defined as *p* < 0.05. Data were presented as mean ± standard deviation. Significant differences were indicated as *p* * < 0.05, *p* ** < 0.01, *p* *** < 0.001, and *p* **** < 0.0001. All plots were visualized using R software (v4.2.2) with the ggplot2 package and associated bioinformatics tools.

A respiratory tract infection model was established using *S. dysgalactiae* F1 (10^9^ CFU/mL) and *S. equi* F4 (10^8^ CFU/mL). At Day 1 post-infection, mice in all infection groups displayed consistent systemic symptoms, including lethargy, sluggish responses, hypoactivity, and ruffled fur. Gross anatomical examination revealed dark red livers and spleens covered with multiple petechiae, as well as pale lungs with petechiae, whereas no visible lesions were found in the heart or intestines. At Day 3, ruffled fur diminished in the single-infection groups (*S. dysgalactiae* F1 and *S. equi* F4), although lethargy and reduced activity persisted. By contrast, no such symptom relief was observed in the co-infection group. Gross pathological lesions resembled those noted at Day 1, except that the co-infection group developed severe pulmonary infection and lung enlargement (Figure 4a). At Day 7, mice in both single-infection groups recovered normal activity, while the co-infection mice still exhibited ruffled fur and sluggish response. Necropsy findings revealed splenomegaly in the *S. equi* F4 single-infection group, whereas the co-infection group presented both hepatosplenomegaly and aggravated pulmonary lesions.

Pulmonary bacterial loads were quantified as follows. For the *S. equi* F4 single-infection group, lung bacterial loads climbed from 1.58 × 10^6^ CFU/g (log10 value = 6.2) at Day 1 to 5.86 × 10^8^ CFU/g (log10 value = 8.77) at Day 3, and further rose to 6.34 × 10^8^ CFU/g (log10 value = 8.80) at Day 7. For the *S. dysgalactiae* F1 single-infection group, loads increased from 5.05 × 10^5^ CFU/g (log10 value = 5.7) at Day 1 to 2.22 × 10^7^ CFU/g (log10 value = 7.3) at Day 3, peaking at 3.94 × 10^7^ CFU/g (log10 value = 7.60) at Day 7. In the co-infection group, total bacterial loads rose from 1.94 × 10^7^ CFU/g (log10 value = 7.3) at Day 1 to 6.06 × 10^8^ CFU/g (log10 value = 8.78) at Day 3, and surged sharply to 3.46 × 10^11^ CFU/g (log10 value = 11.54) at Day 7. No streptococcal colonies were recovered from lung tissue homogenates of healthy control mice (Figure 4b).

Inflammatory cytokine profiles are presented in Figure 4c–g. No significant temporal or intergroup differences in IL-6, IFN-γ, or TNF-α were detected among infected groups, with cytokine concentrations comparable to healthy controls (*p* > 0.05). In contrast, IL-1β concentrations were markedly higher in all infection groups at Days 1, 3, and 7 relative to healthy controls (*p* < 0.05), with no significant variation across infection groups or time points. MCP-1 levels were generally elevated in infected mice versus controls. Specifically, the Day 1 co-infection group and Day 3 *S. equi* F4 single-infection group exhibited significantly higher MCP-1 levels than healthy controls (*p* < 0.05); all other pair-wise comparisons showed no statistical significance.

### 3.7. The Dynamic Transcriptional Landscape of Murine Autoimmune Pathogenesis

**Figure 5:**
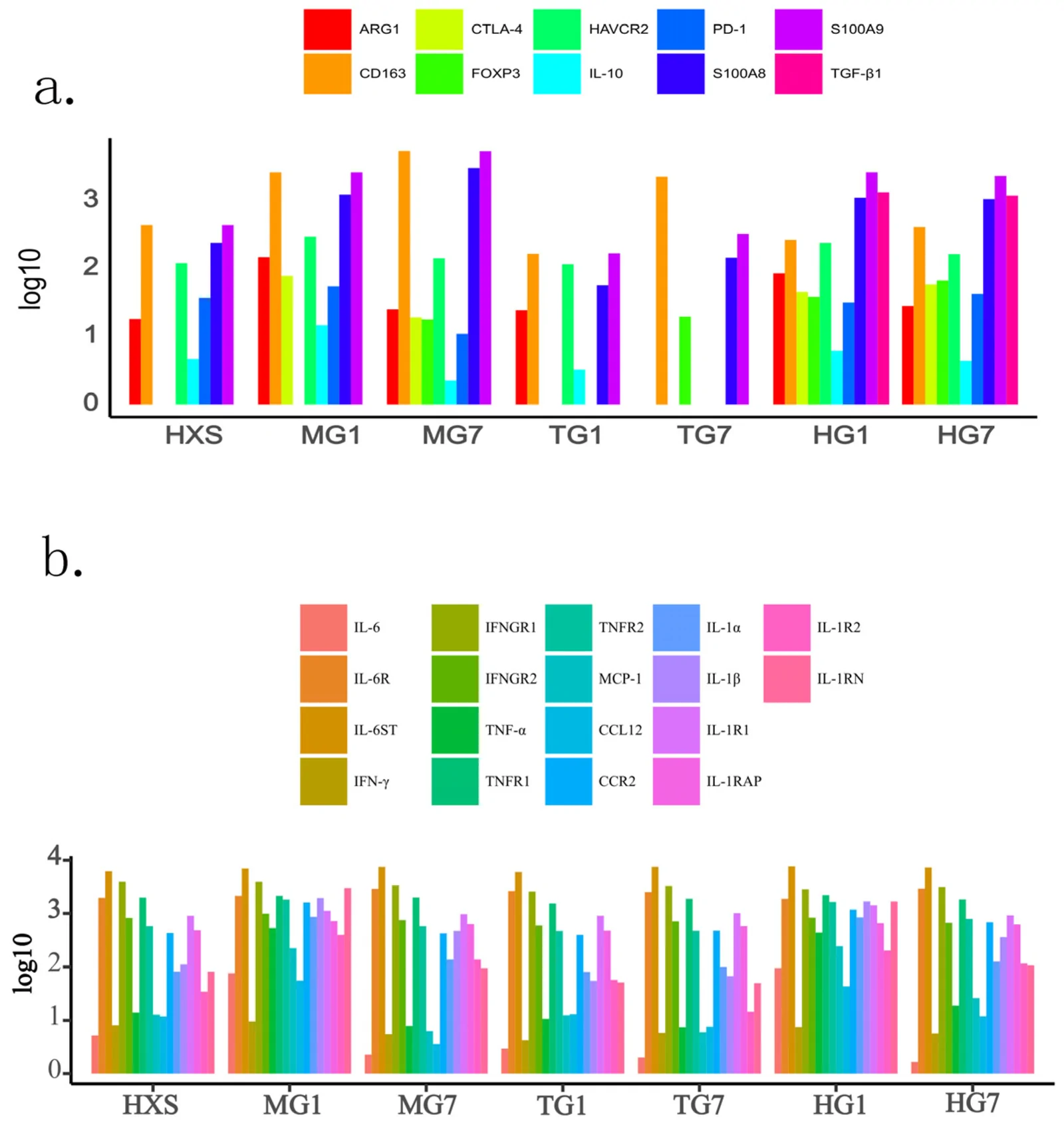
(**a**) immunosuppressive-related molecules, (**b**) immune factors. Transcriptional expression levels of immune genes in mice infected with *S. dysgalactiae*, *S. equi* and their co-infection. Raw read counts were normalized to correct sequencing depth before statistical differential expression analysis. For transcriptomic analysis, three mice with consistent pathological phenotypes and bacterial loads were screened from each group as biological replicates (*n* = 3).

Through transcriptomic analysis, we comprehensively evaluated the expression levels of IL-6, IFN-γ, TNF-α, IL-1β, and MCP-1, as well as their receptor changes, in different infection groups at various infection stages (Figure 5a). In the IL-6 signaling pathway, IL-6 expression was significantly higher in the *S. equi*-infected and mixed-infected groups on Day 1 compared to the healthy control group. However, by Day 7, IL-6 levels in these two groups decreased below the control level, and the changes in IL-6 receptors were not significant across all groups. The expression level of IFN-γ was slightly lower than that of the healthy group in all infected groups except for the *S. equi*-infected group on Day 1. TNF-α was significantly elevated in the *S. equi*-infected and mixed-infected groups on Day 1, and returned to the control level by Day 7 in the mixed-infected group. In other infected groups, TNF-α levels were lower than the control group, and the expression of its receptor TNFR2 was significantly higher than the control group in the *S. equi*-infected and mixed-infected groups on Day 1. MCP-1 (CCL2) expression was increased in the *S. equi*-infected and mixed-infected groups on Day 1, but was significantly lower than the control group in the *S. equi*-infected group on Day 7. CCL12 showed a similar expression trend. The receptor CCR2 of MCP-1 was significantly elevated in the *S. equi*-infected and mixed-infected groups on Day 1. However, on Day 7 of *S. equi* infection, although MCP-1 expression was significantly lower than that on Day 1 and in the control group, the expression level of its receptor CCR2 was consistent with the control group. IL-1β expression was higher than in the control group throughout the infection process in the *S. equi*-infected and mixed-infected groups, and IL-1α showed a similar expression trend. However, there were no significant differences in the expression of IL1R1 and IL1RAP between all infected groups and the control group. The expression trend of IL1R2 was consistent with that of IL-1α and IL-1β, and IL1RN was relatively higher in the *S. equi*-infected and mixed-infected groups on Day 1. Notably, during *S. dysgalactiae* infection, the expression of all cytokines showed no significant increase and was even slightly lower than the control group, and further decreased by Day 7 compared to Day 1.

Compared with the healthy control group, on Day 1 of *S. equi* infection, the expression of ARG1, CD163, HAVCR2, IL10, PD-1, S100A8, and S100A9 was significantly upregulated, while CTLA-4 expression was activated; at Day 7 of infection, ARG1 expression returned to the level of the healthy control group, CD163 continued to increase, CTLA-4 expression was downregulated, HAVCR2 returned to the level of the healthy control group, and the expression of IL-10 and PD-1 was lower than that of the healthy control group, with FOXP3 expression activated (Figure 5b). On Day 1 of *S. dysgalactiae* infection, PD-1 expression was inhibited, and the levels of other cytokines were similar to or lower than those of the healthy control group; at Day 7 of infection, although CD163 expression increased, the expression of multiple cytokines such as ARG1 was inhibited, and FOXP3 expression was activated. On Day 1 of mixed infection, FOXP3 and CTLA-4 expression were activated, and the expression of other cytokines was similar to that of the healthy control group; at Day 7 of infection, the expression of all cytokines did not increase (Figure 5b).

### 3.8. Alterations in Murine Pulmonary Microbiota and Corresponding KEGG Pathway Shifts During Streptococcal Infection

At the genus level, analysis of pulmonary microbiota revealed that co-infection induced a significantly higher abundance of *Streptococcus* compared to other infection groups, whereas mice infected solely with *S. dysgalactiae* exhibited depleted *Streptococcus* populations. Conversely, *Streptococcus* abundance declined progressively over the course of *S. equi* infection.

At Day 1 post-*S. equi* challenge, the relative abundance of *Desulfovibrio* sp., *Enterococcus* sp., *Herbaspirillum* sp., *Sediminibacteria* sp., and *Chlamydia* sp. increased, while those of *Klebsiella* sp. and *Alistipes* sp. Decreased. This microbiota shift coincided with suppressed enrichment of ko04211 (Longevity regulating pathway), ko00311 (Penicillin and cephalosporin biosynthesis), ko05034 (Alcoholism), ko04668 (TNF signaling pathway), ko04911 (Insulin secretion), ko04926 (Relaxin signaling pathway), ko04725 (Cholinergic synapse), and upregulation of ko00281 (Geraniol degradation).

**Figure 6:**
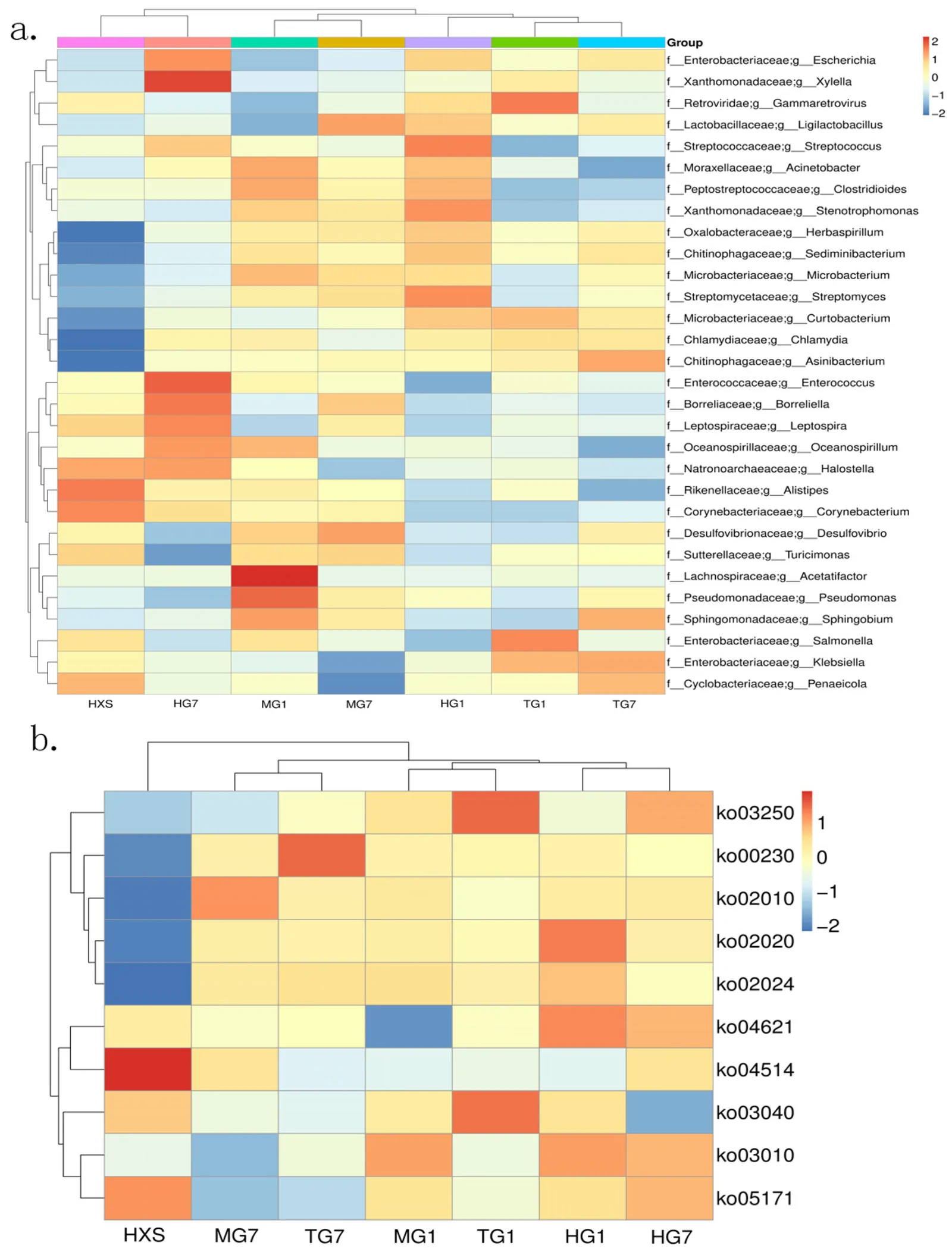
The microbiota abundance and their corresponding KEGG pathway expression profiles in mice infected with *S. dysgalactiae*, *S. equi* and their co-infection. (**a**) The species abundance clustering heatmap. Rows indicate microbial species, columns stand for samples, and the tree on the left displays species clustering results. The color values correspond to row-normalized species relative abundance. (**b**) The clustering heatmap of KEGG-annotated functions. Columns represent samples, rows represent KEGG functional entries, and the left dendrogram reflects functional clustering. Color values are row-normalized pathway relative abundance. For metagenomic analysis, three mice with consistent pathological phenotypes and bacterial loads were screened from each group as biological replicates (*n* = 3).

At Day 7 post-infection, *Desulfovibrio* sp. and *Curtobacterium* sp. were further enriched, while *Klebsiella* sp., *Enterococcus* sp., *Sediminibacteria* sp., *Chlamydia* sp., and *Alistipes* sp. were continuously depleted. Relative to Day 1 samples, this shift triggered elevated enrichment of ko04211 (Longevity regulating pathway), ko04013 (MAPK signaling pathway), and ko00281 (Geraniol degradation), downregulation of ko05225 (Proteoglycans in cancer), and upregulation of ko05034 (Alcoholism), ko04668 (TNF signaling pathway), ko04911 (Insulin secretio), ko04926 (Relaxin signaling pathway), and ko04725 (Cholinergic synapse). Nevertheless, the enrichment status of all these pathways failed to recover to the baseline levels seen in healthy controls (Figure 6a,b).

For mice infected with *S. dysgalactiae* alone at Day 1, *Desulfovibrio* sp., *Turicimonas* sp., and *Alistipes* sp. decreased, while those of *Herbaspirillum* sp., *Sediminibacteria* sp., *Chlamydia* sp., *Curtobacterium* sp., *Escherichia* sp., and *Klebsiella* sp. were enriched. These microbial changes were accompanied by reduced enrichment of eight pathways: ko04211 (Longevity regulating pathway), ko00311 (Penicillin and cephalosporin biosynthesis), ko05034 (Alcoholism), ko04668 (TNF signaling pathway), ko04911 (Insulin secretion), ko04926 (Relaxin signaling pathway), ko04725 (Cholinergic synapse), and ko04013 (MAPK signaling pathway).

At Day 7 post-infection, *Alistipes* sp. remained depleted; *Desulfovibrio* sp. abundance returned to healthy levels, *Herbaspirillum* sp., *Sediminibacteria* sp., and *Escherichia* sp. abundances continued to increase, *Curtobacterium* sp. abundance began to decrease but did not recover to healthy group levels, and *Enterococcus* sp. abundance started to decline, leading to upregulation of ko04211 (Longevity regulating pathway) but not reaching healthy levels, upregulation of ko00311 (Penicillin and cephalosporin biosynthesis) and ko05225 (Proteoglycans in cancer), and continued downregulation of ko00281 (Geraniol degradation) (Figure 6a,b).

In the co-infection group at Day 1, *Desulfovibrio* sp., *Turicimonas* sp., and *Alistipes* sp. decreased, while *Enterococcus* sp., *Herbaspirillum* sp., *Sediminibacteria* sp., *Chlamydia* sp., *Curtobacterium* sp., *Escherichia* sp., and *Klebsiella* sp. increased, concurrent with suppressed enrichment of ko04211 (Longevity regulating pathway), ko05034 (Alcoholism), ko04668 (TNF signaling pathway), ko04911 (Insulin secretion), ko04926 (Relaxin signaling pathway), and ko04725 (Cholinergic synapse).

At Day 7 post co-infection, *Desulfovibrio* sp., *Turicimonas* sp., and *Escherichia* sp. continued to decrease, *Enterococcus* sp. abundance was markedly higher than that in healthy controls; *Alistipes* sp. recovered partially yet remained below baseline; *Herbaspirillum* sp. and *Sediminibacteria* sp. began to decrease but were still higher than healthy levels, This microbial profile resulted in significantly higher enrichment of ko05034 (Alcoholism), ko04668 (TNF signaling pathway), ko04911 (Insulin secretion), ko04926 (Relaxin signaling pathway), and ko04725 (Cholinergic synapse) than in the healthy group, upregulation of ko04211 (Longevity regulating pathway) but not recovering to healthy group levels, downregulation of ko00311 (Penicillin and cephalosporin biosynthesis), continued downregulation of ko04013 (MAPK signaling pathway), and upregulation of ko00281 (Geraniol degradation) but not reaching healthy levels (Figure 6a,b).

## 4. Discussion

This study revealed that at Day 7 following co-infection with *S. dysgalactiae* F1 and *S. equi* F4, the bacterial load in mouse lung tissue reached 10^11^ CFU/g, showing an upward trend compared with single-strain infection groups. Meanwhile, the co-infection group exhibited markedly more severe pathological lesions. *S. equi* F4 exhibited a β-hemolytic (complete hemolysis) phenotype; nevertheless, in vitro co-culture assays detected no synergistic enhancement of hemolytic activity between the two isolates. Crystal violet staining-based biofilm quantification demonstrated that strain F1 possessed strong biofilm-forming capacity, whereas all other culture groups showed weak biofilm production. The total biofilm biomass under co-cultivation was lower than that of strain F1 cultured alone. Consistently, strain F1 generated abundant biofilms under in vitro culture but exerted weaker pathogenic effects during single-strain infection in vivo. This apparent phenotypic discrepancy stemmed from fundamental differences between simplified laboratory culture conditions and the complex host microenvironment. In vitro co-cultures lacked host inflammatory cues, tissue matrices, and sustained nutrient resources; thus, the two isolates competed for limited nutrients, which suppressed the biofilm production of strain F1. In vivo, hemolytic *S. equi* F4 induced severe pulmonary epithelial damage and remodeled local immune transcriptional profiles, potentially forming a permissive inflammatory niche that facilitated massive bacterial colonization. These effects collectively elevated the pulmonary bacterial loads of both strains, resulting in a peak bacterial burden of 10^11^ CFU/g in the co-infection group.

Analysis of the dynamic expression of immune and immunosuppressive factors demonstrated that *S. dysgalactiae* F1 exhibits an inherently low-inflammatory phenotype during pulmonary infection. Following single infection with F1, the transcriptional levels of host pro-inflammatory immune factors (e.g., IL-6, TNF-α) and immunosuppressive mediators (e.g., ARG1, CD163, and FOXP3) were considerably lower than those observed in the *S. equi* F4 single-infection and co-infection groups. This muted immune response mainly occurs at the early infection stage, which likely represents a passive low-recognition characteristic rather than an active immune suppression strategy [18,19].

Consistent with this phenotypic feature, genome-wide virulence factor prediction based on the VFDB revealed that *S. dysgalactiae* F1 lacks prominent homologous sequences of classic streptococcal immune escape factors, including endoglycosidase and *S. dysgalactiae* exotoxin G [20,21]. Notably, these in silico predictions rely on sequence alignment and cannot fully exclude the existence of unannotated or functionally divergent virulence-related homologs. Nevertheless, multiple adhesion-associated proteins, such as fibronectin-binding protein and laminin-binding protein, were annotated in the F1 genome, which may support its colonization within alveolar tissues. In addition, metabolism-related proteins, including trehalose-recycling ABC transporter and glutamine synthetase, may assist F1 in adapting to and surviving in the host microenvironment [22,23,24,25,26,27,28].

In contrast, infection with *S. equi* F4 (MG1, MG7) significantly upregulated both pro-inflammatory and immunosuppressive gene expression. Notably, markedly elevated mRNA levels of IL-6 and TNF-α were detected at Day 1 post-F4 single infection, implying that the robust hemolytic activity of F4 may provoke intense acute pulmonary inflammation [29]. In the co-infection context, excessive inflammation triggered by F4 may gradually drive host immunity toward an immune exhaustion-like phenotype, as indicated by the sustained transcriptional upregulation of immunosuppressive markers (ARG1, CD163, and FOXP3) at Day 7. F4-mediated tissue injury and alveolar barrier disruption may further provide favorable niches for F1 colonization via its adhesion-related proteins. Given the limited intrinsic immune evasion capacity of F1, its in vivo persistence and proliferation are presumably reliant on the immunosuppressive niche induced by F4, a process potentially accompanied by reduced antibody production and impaired effector T cell function. A progressive shift toward an immunosuppressive state may impair pulmonary bacterial clearance and facilitate uncontrolled proliferation of *S. dysgalactiae* F1 in co-infected mice, yet this mechanistic speculation awaits further functional validation. Such synergistic interactions may partially explain the remarkably high bacterial load (10^11^ CFU/g) detected at the late stage of co-infection. Furthermore, the type VII secretion system of F4 may disrupt host epithelial barriers through secreted effector proteins, which could indirectly enhance the tissue colonization and outgrowth of co-infecting *S. dysgalactiae* F1 [30].

Notably, there was inconsistency between ELISA and transcriptome sequencing results in the expression of some cytokines. ELISA results showed no significant differences in the protein levels of IL-6, IFN-γ, and TNF-α among the infection groups, while transcriptome analysis revealed significantly elevated mRNA levels of IL-6 and TNF-α in the *S. equi* single-infection group and co-infection group on Day 1. This discrepancy may be attributed to the complexity of post-transcriptional regulatory mechanisms—strong early transcriptional activation failed to be fully converted into stable protein accumulation, or it reflects the shorter half-life of proteins compared to mRNA, resulting in no significant difference in protein levels despite upregulated transcription [31].

Lung tissue represents a typical low-biomass matrix and is prone to exogenous microbial contamination at every stage of the metagenomic sequencing workflow. Given the extremely low baseline microbial load in mouse lungs, uninfected healthy lung samples were utilized as endogenous background controls to distinguish *Streptococcus*-derived microbial signals from the indigenous pulmonary microbiota, in lieu of conventional extraction blanks and environmental controls. Furthermore, no dedicated bioinformatic decontamination pipelines were adopted in this study. After filtering out mouse host genomic sequences, raw sequencing reads were directly processed for taxonomic classification and functional pathway analysis, without algorithmic filtering of sequences annotated as common laboratory contaminants. The absence of physical negative controls and supplementary computational decontamination inevitably retained trace exogenous environmental contaminants within the sequencing dataset, which constituted a major inherent limitation of the present analysis. Despite this limitation, clear differential abundance patterns were detected: the relative abundance of *Streptococcus* in the co-infection group was markedly higher than that in all single-strain infection groups. Notably, the *S. dysgalactiae* monoinfection group displayed the lowest *Streptococcus* proportion, whereas *Streptococcus* abundance declined progressively over time in the *S. equi* monoinfection group. Additional microbiota analysis demonstrated that early infection induced an elevation in *Desulfovibrio* abundance exclusively in the *S. equi* single-infection group. In contrast, this taxon was depleted in both the *S. dysgalactiae* single-infection and co-infection groups. On Day 7 post-infection, *Desulfovibrio* levels continued to increase in the *S. equi* group and rebounded in the *S. dysgalactiae* group, while sustained depletion was observed in co-infected lungs.

KEGG functional annotation revealed significant enrichment of alcohol metabolic pathways in the pulmonary microbiota of co-infected mice. Importantly, although this pathway was classified under human disease-related entries in the KEGG database, such annotation merely indicates the presence of homologous functional genes within the microbiota. It cannot directly confirm the production of alcohol metabolites in lung tissue nor validate the actual catalytic activity of the corresponding enzymes in vivo. We therefore hypothesize that shifts in the composition of the microbial community may regulate short-chain alcohol metabolism in lung tissue, and targeted metabolomic profiling assays are required to validate this hypothesis.

Several limitations of this study should be noted. First, mouse lung tissues from each group were pooled for bacterial load quantification, which precluded independent analysis of individual sample variation and may conceal potential outlier data, limiting the reliability of statistical evaluation in this study. Accordingly, intergroup differences in bacterial CFU were only analyzed descriptively, and the observed trend of elevated bacterial load in co-infected mice failed to reach statistical significance. To address this limitation and obtain robust, statistically verifiable data, individual lung tissue sampling and independent biological replicate analysis will be adopted in our future investigations. Second, all infection experiments were conducted on BALB/c intranasal mouse models instead of horses, the natural host. Marked interspecies differences in immune responses and pathogen susceptibility mean the pathogenic characteristics observed here need further verification in equine models. Third, all strains were isolated from a single diseased foal, limiting sample representativeness. Pathological phenotypes obtained under controlled laboratory conditions cannot support broad epidemiological inferences about streptococcal co-infection in horse herds. Future research will include multiple clinical isolates from different horses and field epidemiological surveys to validate the generalizability of our findings. Fourth, high bacterial inocula were applied in this study because sublethal doses produced mild and indistinguishable lung damage, making it difficult to identify specific synergistic virulence. The current lethal-dose setup enables clear discrimination of synergistic pathogenic effects from simple additive tissue injury. The 10:1 ratio of *S. equi* to *S. dysgalactiae* was set based on respective LD_50_ values to balance strain lethality. Future work will adopt sublethal doses, multiple strain ratios, and clinically relevant proportions to better mimic natural co-infection. Fifth, the simultaneous dual-strain nasal challenge adopted herein prevents rigorous validation of our sequential pathogenic cascade hypothesis. We hypothesize that primary *S. equi* infection may trigger pulmonary inflammation and immune dysfunction to establish an immunosuppressive microenvironment, which allows robust proliferation of *S. dysgalactiae*. A staggered infection model with a 1–2-day interval between the two strains will be utilized in follow-up studies to confirm this temporal causal relationship. Sixth, the number of mouse samples in this study was limited, and all lung tissues were used for transcriptome sequencing, metagenomic sequencing and bacterial load quantification. Accordingly, only macroscopic morphological observations of lung tissues were presented in the original manuscript, with no residual tissues left for histopathological section preparation and microscopic examination. In future research, comprehensive histopathological staining combined with semi-quantitative scoring will be performed to further validate tissue injury phenotypes and inflammatory lesions at the microscopic level.

## 5. Conclusions

In conclusion, based on a mouse pulmonary infection model, this study systematically compared the differences in lung pathological damage, bacterial colonization level, host immune response, and lung microbiota structure between single infection and co-infection with *S. equi* and *S. dysgalactiae*. The biofilm biomass of *S. dysgalactiae* F1 was higher than that of all other groups. The results demonstrated that co-infection with the two streptococcal strains led to elevated bacterial colonization in mouse lung tissues, aggravated macroscopic lung lesions, and induced disordered host immune responses and lung microbiota imbalance. This study preliminarily clarified the variation characteristics of various indicators in mice under co-infection compared with single infection, providing a reliable experimental basis and valuable reference for further exploring the pathogenic mechanism of streptococcal co-infection and optimizing relevant clinical intervention strategies.

## Figures and Tables

**Table 1:** The diameter of the inhibition zone around *S. dysgalactiae* and *S. equi*.

K-B Disk	*S. dysgalactiae* F1	*S. dysgalactiae* F2	*S. dysgalactiae* F3	*S. equi* F4	*S. equi* F5	ATCC49619
AK (30 μg)	9.80 ± 0.08 mm	10.15 ± 0.51 mm	10.65 ± 0.15 mm	7.83 ± 0.82 mm	7.00 ± 0.00 mm	18.79 ± 0.00 mm
CIP (5 μg)	23.05 ± 0.18 mm	21.32 ± 0.38 mm	21.28 ± 0.08 mm	22.80 ± 0.45 mm	22.57 ± 0.30 mm	48.70 ± 0.01 mm
FOT (200 μg)	25.01 ± 0.04 mm	22.16 ± 0.83 mm	21.86 ± 0.22 mm	30.23 ± 0.53 mm	31.20 ± 0.08 mm	43.32 ± 0.20 mm
TGC (15 μg)	24.55 ± 0.32 mm	25.16 ± 0.21 mm	24.16 ± 0.13 mm	25.72 ± 0.58 mm	25.24 ± 1.06 mm	37.32 ± 0.02 mm
AMP (10 μg)	36.91 ± 0.40 mm	33.31 ± 0.56 mm	32.00 ± 0.23 mm	34.47 ± 1.98 mm	34.21 ± 0.78 mm	32.51 ± 0.02 mm
CN (10 μg)	17.51 ± 0.50 mm	16.47 ± 0.02 mm	18.18 ± 0.79 mm	14.927 ± 0.38 mm	14.76 ± 0.05 mm	15.12 ± 0.11 mm
SXT (25 μg)	28.48 ± 0.95 mm	24.53 ± 0.03 mm	24.52 ± 0.33 mm	28.75 ± 1.41 mm	30.17 ± 0.42 mm	28.11 ± 0.14 mm
TE (30 μg)	22.61 ± 0.38 mm	22.78 ± 0.56 mm	23.24 ± 0.48 mm	20.875 ± 0.83 mm	20.99 ± 0.23 mm	40.32 ± 0.10 mm
MEM (10 μg)	38.71 ± 0.40 mm	35.43 ± 0.44 mm	30.57 ± 0.36 mm	34.58 ± 0.16 mm	35.81 ± 0.03 mm	30.11 ± 0.14 mm
CTX (30 μg)	38.68 ± 0.33 mm	35.41 ± 0.27 mm	32.13 ± 0.15 mm	35.60 ± 0.02 mm	36.68 ± 0.11 mm	38.59 ± 0.21 nm
C (30 μg)	25.35 ± 0.37 mm	24.99 ± 0.01 mm	23.10 ± 0.12 mm	27.41 ± 0.41 mm	26.24 ± 0.37 mm	25.17 ± 0.11 nm

## Data Availability

The complete sequence of *S. dysgalactiae* isolates (F1, F2, F3) and *S. equi* (F4 and F5) has been submitted to GenBank under the accession numbers JBWKAG000000000, JBWKAI000000000, JBWKAH000000000, JBWKAE000000000, and JBWKAF000000000. Transcriptome and metagenome data derived from the lungs of mice in different infection groups at distinct post-infection stages have been uploaded to the CNCB (China National Center for Bioinformatics) (https://ngdc.cncb.ac.cn/gsub accessed on 20 January 2026) under accession numbers CRA037801 and CRA038013.

## Supplementary Materials

The following supporting information can be downloaded at: https://www.mdpi.com/article/10.3390/microorganisms14081674/s1, Table S1: Strain information for *S. dysgalactiae* and *S. equi* from GenBank; Table S2: 16S rRNA sequences of *Streptococcus dysgalactiae* and *Streptococcus equi* in this study; Table S3: Sequencing quality control information of *Streptococcus dysgalactiae* and *Streptococcus equi* in this study; Table S4: Multilocus Sequence Typing of Streptococcus dysgalactiae and Streptococcus equi in this study; Table S5: Screening results of *S. dysgalactiae* and *S. equi* against VFDB and CARD in this study.
